# Common osteoporosis drug associated with increased rates of depression and anxiety

**DOI:** 10.1038/s41598-021-03214-x

**Published:** 2021-12-14

**Authors:** Dro Keshishi, Tigran Makunts, Ruben Abagyan

**Affiliations:** 1 Skaggs School of Pharmacy and Pharmaceutical Sciences, University of California San Diego, San Diego, CA USA; 2 Oak Ridge Institute of Science and Education Fellowship, Center for Drug Evaluation and Research, United States Food and Drug Administration, Silver Spring, MD USA

**Keywords:** Osteoporosis, Adverse effects, Bisphosphonates, Anxiety, Depression

## Abstract

Osteoporosis affects over 10 million Americans over 50. Bisphosphonate therapy, mainly alendronate, is amongst the most prescribed treatments for the disease. The use of alendronate and other bisphosphonates has been associated with depressive symptoms in recent case reports. In this study we quantified this association by analyzing over 100,000 adverse events reports from the Food and Drug Administration Adverse Events Reporting System (FAERS) and the World Health Organization’s (WHO) global database for adverse drug reactions, ADRs, VigiAccess. We found that alendronate therapy is significantly associated with depression and anxiety when compared to other first-line osteoporosis treatments. The reported risk of depressive ADRs was found to be over 14-fold greater in patients taking alendronate under the age of 65 and over fourfold greater for patients over 65 compared to the control. Several hypotheses concerning the molecular mechanism of the observed association of alendronate and depressive symptoms were discussed.

## Introduction

In 2014, the National Osteoporosis Foundation estimated that 10.2 million Americans suffer from osteoporosis, and 43.4 million have low bone mineral density. With the expected growth of the aging population this number is likely to increase in the future^1^.

Pharmacological therapies used to improve patient bone mineral density reduce the risk of fractures and increase the overall quality of life. Amongst the most common drug therapies for osteoporosis, as recommended by the American College of Physicians, are bisphosphonates, including alendronate, zoledronate, ibandronate and risedronate. Other first-line options with a different molecular mechanism include denosumab and teriparatide^2^. Bisphosphonates are small molecule drugs that bind to hydroxyapatite in the bone and lead to inhibition of osteoclasts^3^. Denosumab, which is a fully humanized monoclonal antibody, acts by inhibiting receptor activator of nuclear factor kappa-Β ligand, and leads to lower bone turnover, therefore increasing bone mineral density^4^. In contrast, teriparatide is a synthetic analog of parathyroid hormone which stimulates osteoblast activity and bone formation^5^. The most common recognized side effects of *oral* bisphosphonates include upset stomach, acid reflux, esophagitis and ulcerations^6–8^. *Intravenously* administered bisphosphonates, ibandronate and zoledronate, are associated with injection site reactions^8,9^. Subcutaneous injection of denosumab is commonly accompanied with back pain, arthralgia, pain in extremity, cystitis, and nasopharyngitis^10^. Teriparatide, also administered subcutaneously, is associated with injection site pain, nausea, dizziness, and headaches^11^.

Previous studies have established an association between depression and osteoporosis, with depression being the predictor of the disease state^12^. At the same time, a diagnosis of osteoporosis itself can potentially lead to depression due to decreased mobility and overall quality of life^13^. A 2010 report demonstrated an association between the bisphosphonates as a class and depressive symptoms in humans, reporting a reported odds ratio (ROR) of 2.3 (2.1–2.5) in WHO database, while in was not significant for alendronate 1.3 (0.7–2.5) in the Lareb database^14^. Interestingly, a longitudinal study of osteoporosis patients revealed that anxiety was as an independent predictor of bone mineral density^15^.

In our study, we take a closer look at each individual bisphosphonate, and evaluate their individual ADR associations by analyzing the osteoporosis subset of over 15 million post marketing adverse drug event reports in the FAERS database. We evaluated the ROR magnitude and 95% confidence intervals in males and female patients with only osteoporosis and only taking one pharmaceutical agent. To confirm the conclusions, we perform a similar analysis of the World Health Organization global database for adverse drug reactions.

## Methods

Data sets were obtained from the FAERS/AERS reports and are available to the public online via: http://www.fda.gov/Drugs/GuidanceComplianceRegulatoryInformation/Surveillance/AdverseDrugEffects/ucm082193.htm.

 FAERS contains voluntary submissions of medication-related adverse events from patients, healthcare providers, and legal representatives to the FDA through MedWatch. Additionally, reports collected by the pharmaceutical company pharmacovigilance teams are mandated to be forwarded to the FDA.

Quarterly reports were downloaded in .TXT format and modified to a table field structure^16–18^. At the time of the study the FAERS database contained over 15 million adverse event reports compiled quarterly from January 2004 to December 2020. In this study, reports of patients with exclusively osteoporosis indication were selected to reduce indication based confounding effects, resulting in 139,093 reports. Of these reports, only monotherapy reports were selected. Monotherapy reports were selected for the analysis to avoid confounding effects by other drugs and potential drug interactions. Alendronate, zoledronate, risedronate, ibandronate, denosumab and teriparatide were recommended first-line therapies based on the Endocrine Society^19^, the American Academy of Family Physicians^20^, the National Osteoporosis Foundation^21^ guidelines. Teriparatide was selected as the control due to two criteria: (i) its mechanism of action being distinct form bisphosphonates and denosumab, (ii) large number or reports necessary to ensure statistical significance of the results. Monotherapy cohorts for alendronate (n = 7,821), zoledronate (n = 9,367), risedronate (n = 1,168), ibandronate (n = 3,727), denosumab (n = 15,812) and teriparatide (n = 45,052) were further split by age into groups under and over 65 to evaluate the potential impact of age on the reported frequency of adverse events (Table 1). The choice for a single cut-off age between the groups is always somewhat arbitrary, given the gradual continuous decline of the bone mass density, BMD. However dividing patients into 2 groups is convenient for the analysis, and we based our choice of 65 on the plateauing of the BMD decline plot in female patients. Demographic analysis was performed to support the cohort selection process (Table 2).

**Table 1:** Patient sex demographics in FAERS drug cohorts.

Sex	Alendronate	Zoledronate	Risedronate	Ibandronate	Denosumab	Teriparatide
Female	6,540	7,997	1,051	3,431	13,242	40,821
Male	757	1,145	57	199	1,759	3,974
Unreported	524	225	60	97	811	257
Female frequency (%)	83.6	85.3	89.9	92.1	83.7	90.6
Male frequency (%)	9.6	12.2	4.8	5.3	11.1	8.8

**Table 2:** Patient reported age in FAERS drug cohorts.

Age	Alendronate	Zoledronate	Risedronate	Ibandronate	Denosumab	Teriparatide
Mean age (SD)	68.6 (11.9)	70.3 (12.9)	67.5 (12.1)	68.6 (12.3)	73.8 (11.5)	70.9 (11.3)
Unreported (%)	40.0	33.8	29.3	34.2	30.4	40.6

### Study outcomes

Depression or depressive disorder related adverse events were chosen as the outcome of interest and defined in FAERS by the following MedDRA (Medical Dictionary for Regulatory Activities) terms: *depression, treatment resistant depression, depressed mood, major depression, depressive symptom, agitated depression, persistent depressive disorder, depression suicidal, suicidal ideation, suicide attempt,* and *suicidal behavior*^22^. Similarly, the outcome of anxiety was defined in FAERS by the following MedDRA terms: *anxiety, anxiety disorder, generalized anxiety, anxiety state, anxious mood,* and *anxiety aggravated*.

Additional data was obtained from VigiAccess via the following webpage which is available publicly: http://www.vigiaccess.org.

The database contains suspected adverse reactions from drug authorities of over 110 different countries. The cohorts were similarly selected based on drug therapy with alendronate (n = 51,022), zoledronate (n = 54,018), risedronate (n = 13,507), ibandronate (n = 25,575), denosumab (n = 129,045), and teriparatide (n = 140,664). Unlike FAERS, the data download option was not available, and we used http://www.vigiaccess.org/ online interface. Statistical analysis was performed based on relative reported frequencies, along with 95% confidence intervals of the ROR values.

### Data availability

FAERS/AERS datasets are available to the public online: https://www.fda.gov/drugs/questions-and-answers-fdas-adverse-event-reporting-system-faers/fda-adverse-event-reporting-system-faers-latest-quarterly-data-files and http://www.vigiaccess.org/.

### Statistical analysis

RORs were calculated using the following equations:

$$\begin{aligned} {\mathrm{ROR}} & = ({\mathrm{a}}/{\mathrm{b}})/({\mathrm{c}}/{\mathrm{d}}) \\ {\mathrm{LnROR}} & = {\mathrm{Ln}}({\mathrm{ROR}}) \\ \end{aligned}$$

Standard Error of Log Reporting Odds Ratio:

$$\begin{aligned} & {\mathrm{SELnROR}} = \sqrt {\left( {1/a + 1/b + 1/c + 1/d} \right)} \\ & \quad 95\% \,{\mathrm{Confidence}}\,{\mathrm{Interval}} = \left[ { e^{{\left( {LnROR - 1.96*SELnROR} \right)}} , e^{{\left( {LnROR + 1.96*SELnROR} \right)}} } \right] \\ \end{aligned}$$

where

- Depression/Anxiety cases in the exposed group with an AE
- Cases in the exposed group with no AE
- Depression/Anxiety cases in the control group with the AE
- Cases in the control group with no AE.

### Ethics statement

There was no direct human participation in this study. The study used de-identified datasets. Institutional Review Board requirements do not apply under 45 CFR 46.102. All experiments were performed in accordance with relevant guidelines and regulations.

## Results

### Reported statistics of anxiety and depression in patients under 65 in FAERS

**Figure 1:**
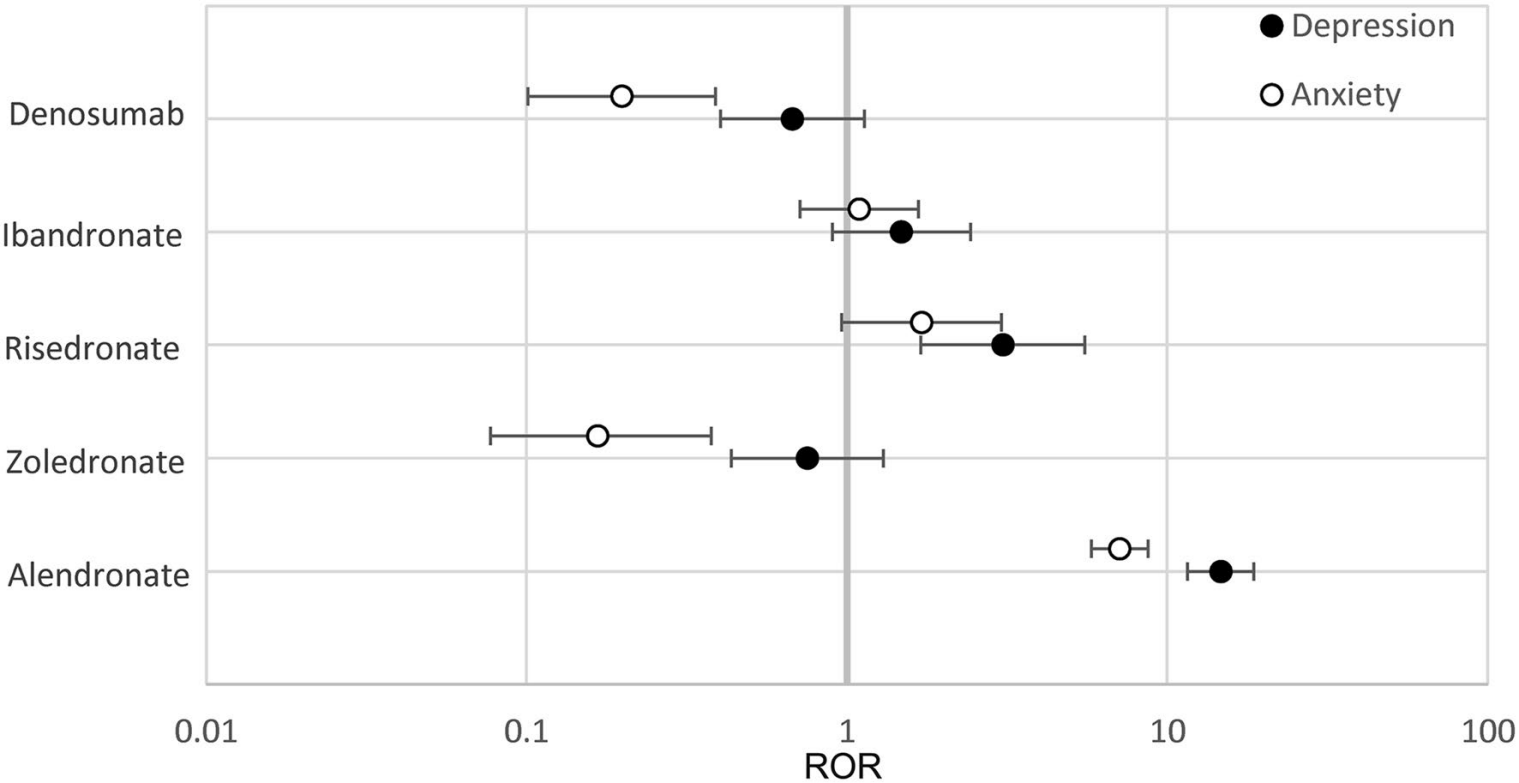
RORs of Depression and Anxiety for ≤ 65 years. Reporting odds ratios were calculated for the frequencies of depression and anxiety in patients ≤ 65 years. The ranges depicted are indicative of 95% confidence intervals, the X-axis is presented in logarithmic scale.

In osteoporosis patients 65 years or below on bisphosphonate monotherapy, ROR values were calculated based on reported frequencies (Tables 3 and 4, Fig. 1). *Alendronate* had a significantly higher odds ratio of both depression and anxiety (ROR 14.67, 95% CI [11.55–18.63], ROR 7.10, 95% CI [5.79–8.71]) for depression and anxiety respectively. Risedronate was the only other treatment to show an increase in odds ratio, however only for the depression outcome and at a lower magnitude than alendronate (3.06 [1.70–5.52]). In contrast, zoledronate and denosumab showed a statistically significant decrease of anxiety (0.167 [0.074–0.38]) and (0.20 [0.10–0.39]) respectively (Table 4, Fig. 1). While estimated RORs for the other therapeutics did not reach statistical significance when the 95% CI was taken into consideration.

**Table 3:** Reported frequencies of depression and anxiety AEs in cohorts.

	Alendronate	Zoledronate	Risedronate	Ibandronate	Denosumab	Teriparatide
≤ 65 years depression (%)	265/1,627 (16.3)	15/1,519 (0.98)	13/333 (3.9)	19/988 (1.9)	17/1,916 (0.89)	98/7,489 (1.3)
≤ 65 years anxiety (%)	235/1,627 (14.4)	6/1,519 (0.39)	13/333 (3.9)	25/988 (2.5)	9/1,916 (0.47)	174/7,489 (2.3)
> 65 years depression (%)	100/2,543 (3.9)	36/3,317 (1.1)	7/437 (1.6)	19/1,574 (1.2)	51/7,292 (0.70)	206/18,491 (1.1)
> 65years anxiety (%)	110/2,543 (4.3)	21/3,317 (0.63)	12/437 (2.7)	8/1,574 (0.51)	37/7,292 (0.51)	359/18,491 (1.94)

**Table 4:** RORs for depression and anxiety ages ≤ 65 years.

	Alendronate	Zoledronate	Risedronate	Ibandronate	Denosumab
Depression ROR (95% CI)	**14.67 (11.55–18.63)**	0.75 (0.44–1.30)	**3.06 (1.70–5.52)**	1.48 (0.90–2.43)	0.68 (0.40–1.13)
Anxiety ROR (95% CI)	**7.10 (5.79–8.71)**	*0.167 (0.074–0.38)*	1.71 (0.96–3.03)	1.09 (0.71–1.67)	*0.20 (0.10–0.39)*

Significant values are in bold for positive and italic for negative correlations respectively.

### Reported statistics of anxiety and depression in patients over 65 years old in FAERS

**Figure 2:**
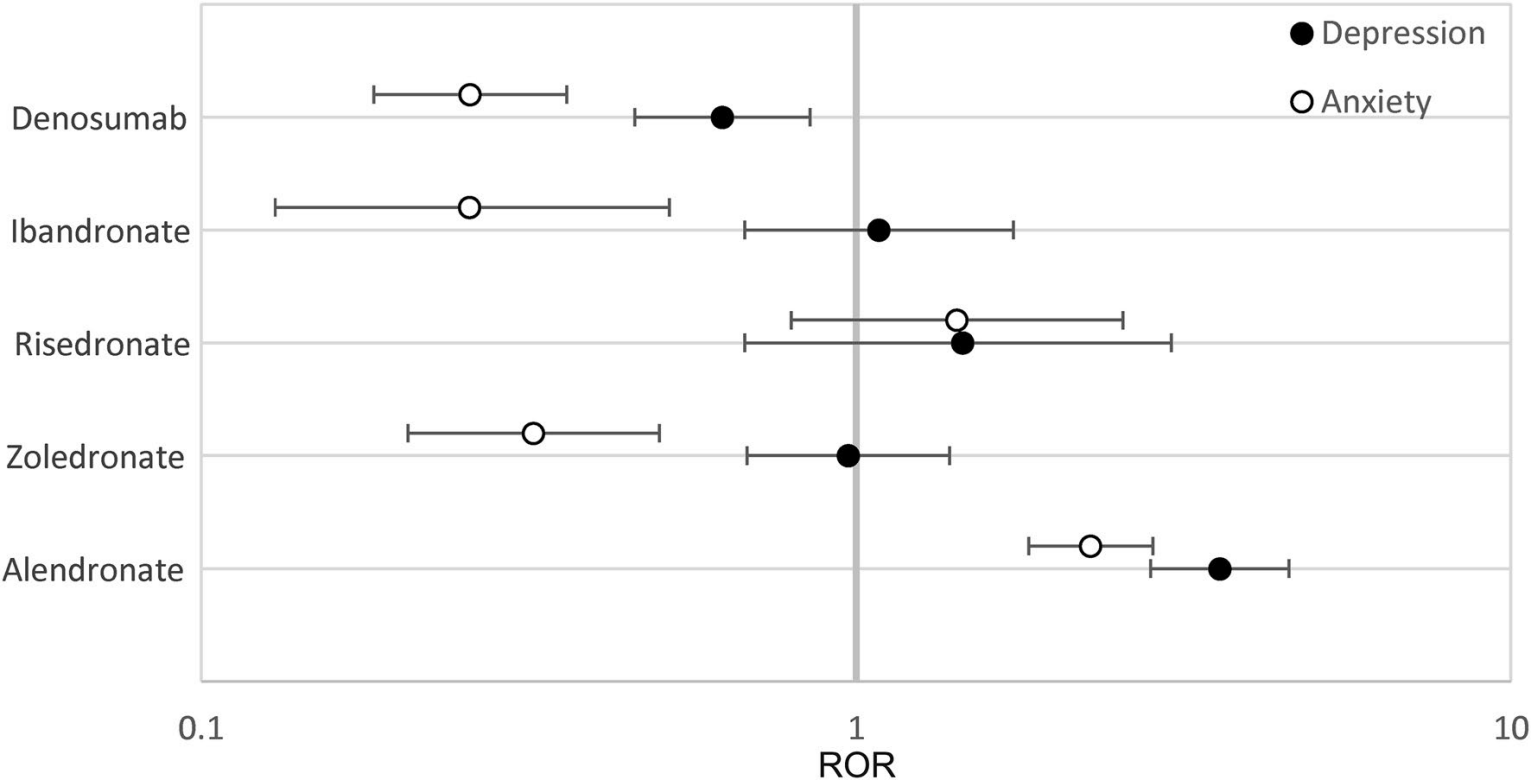
RORs of Depression and Anxiety for ages over 65 years old. Reporting odds ratios relative to teriparatide as an active control were calculated for the frequencies of depression and anxiety as an adverse event in patients over 65. The ranges depicted are indicative of 95% confidence intervals, the X-axis is presented in logarithmic scale.

In subjects over 65, alendronate was the only treatment that had significantly elevated reported odds ratio of depression and anxiety in the cohorts (3.60 [2.82–4.59], 2.28 [1.84–2.84]) compared to the control, teriparatide (Table 5, Fig. 2). In contrast, zoledronate, ibandronate and denosumab showed significant decreases in the odds ratio of anxiety in patients over the age of 65 (0.32 [0.21–0.50]), (0.26 [0.13–0.52]) and (0.26 [0.18–0.36]) compared to the same control (Table 5, Fig. 2).

**Table 5:** RORs for depression and anxiety AEs over 65 years old.

	Alendronate	Zoledronate	Risedronate	Ibandronate	Denosumab
Depression ROR (95% CI)	**3.60 (2.82–4.59)**	0.97 (0.68–1.39)	1.45 (0.68–3.08)	1.08 (0.68–1.74)	*0.63 (0.46–0.85)*
Anxiety ROR (95%CI)	**2.28 (1.84–2.84)**	*0.32 (0.21–0.50)*	1.43 (0.80–2.56)	*0.26 (0.13–0.52)*	*0.26 (0.18–0.36)*

Significant values are in bold for positive and italic for negative correlations respectively.

### WHO Uppsala Centre for international data monitoring, VigiAccess database

Adverse events data from the VigiAccess webpage, was used to confirm the observed association in FAERS using a different data source. Reporting odds ratios were calculated for the frequencies of depression and anxiety compared to teriparatide as defined before. We used the database’s online interface to define the drug names and parameters of the analysis.

**Figure 3:**
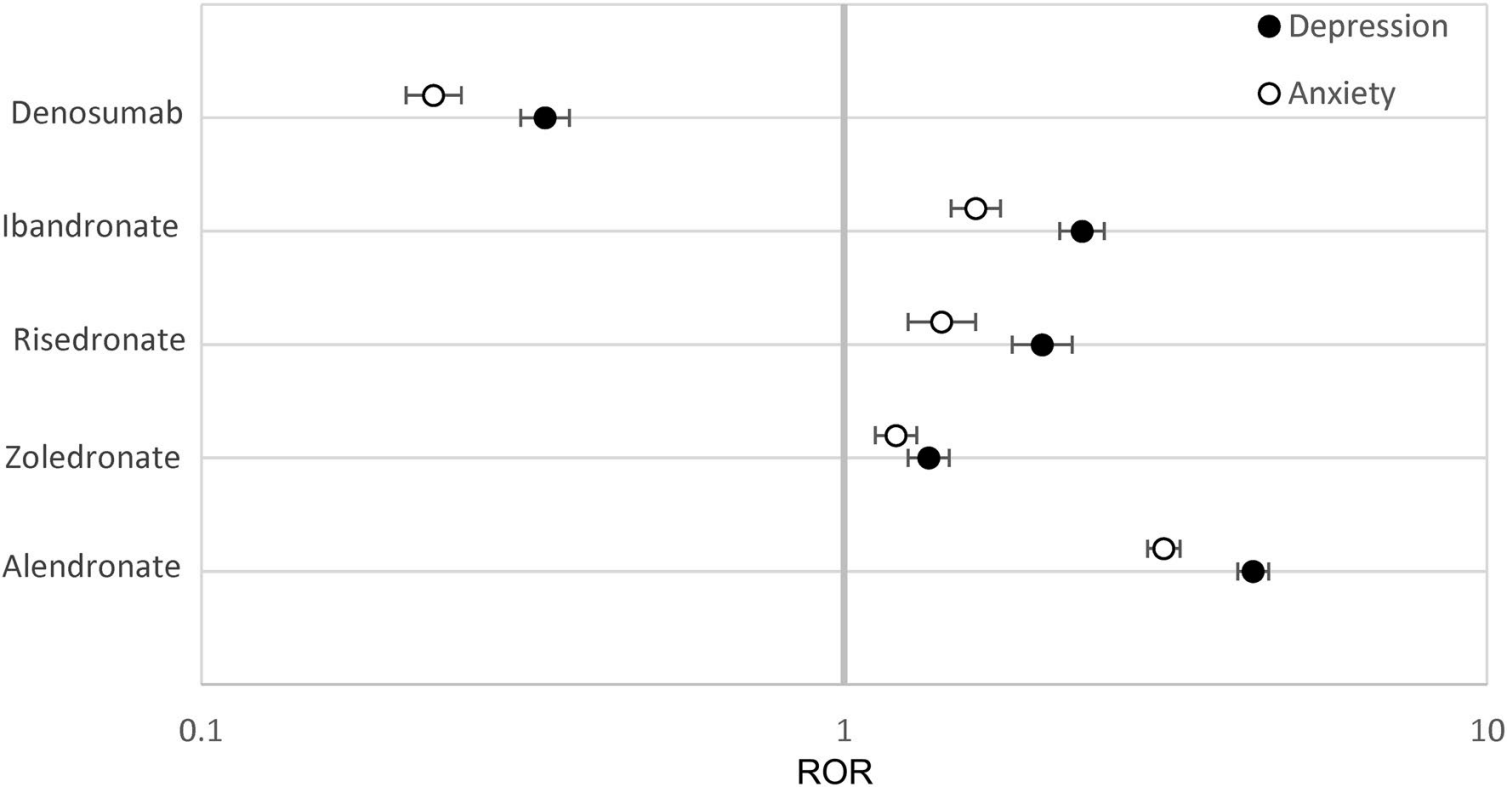
RORs for Depression and Anxiety in VigiAccess, all ages. Reporting odds ratios relative to teriparatide as an active control were calculated for the frequencies of depression or anxiety as an adverse event in patients. The ranges depicted in the forest plot are indicative of 95% confidence intervals and the X-axis is presented in logarithmic scale.

The bisphosphonates as a class demonstrated significantly higher reporting odds ratios of both depression and anxiety with alendronate being of the highest magnitude, (4.33 [4.09–4.58], 3.14 [2.97–3.33]) for depression and anxiety respectively, in agreement with our FAERS analysis. Ibandronate had a weaker ADR association (2.34 [2.17–2.54], 1.60 [1.47–1.75]) for depression and anxiety respectively, followed by risedronate (2.03 [1.82–2.26], 1.42 [1.26–1.60]). Zoledronate had the lowest odds ratio within the bisphosphonate class (1.35 [1.26–1.46], 1.20 [1.12–1.30]) for depression and anxiety respectively, and denosumab had a significantly lower reporting odds ratio of depression and anxiety with (0.34 [0.31–0.37], 0.23 [0.21–0.25]) for depression and anxiety respectively (Tables 6 and 7, Fig. 3).

**Table 6:** Frequencies of depression and anxiety AEs, VigiAccess data.

	Alendronate	Zoledronate	Risedronate	Ibandronate	Denosumab	Teriparatide
Depression (%)	3180/51,022 (6.23)	1101/54,018 (2.04)	409/13,507 (3.03)	889/25,575 (3.48)	676/129,045 (0.52)	2128/140,664 (1.5)
Anxiety (%)	2477/51,022 (4.85)	1036/54,018 (1.92)	304/13,507 (2.25)	648/25,575 (2.53)	480/129,045 (0.37)	2247/140,664 (1.60)

**Table 7:** RORs for depression and anxiety, VigiAccess data.

	Alendronate	Zoledronate	Risedronate	Ibandronate	Denosumab
Depression ROR(95% CI)	4.33 (4.09–4.58)	1.35 (1.26–1.46)	2.03 (1.82–2.26)	2.34 (2.17–2.54)	0.34 (0.31–0.37)
Anxiety ROR (95%CI)	3.14 (2.97–3.33)	1.20 (1.12–1.30)	1.42 (1.26–1.60)	1.60 (1.47–1.75)	0.23 (0.21–0.25)

It’s noteworthy to mention the given the larger size of the WHO database, the weaker associations in FAERS analysis become more statistically significant in VigiAccess, with alendronate remaining as the most depression and anxiety associated bisphosphonate.

Additionally, it is important to keep in mind that the numbers and the signal strength are not directly comparable between FAERS and WHO database results due to the different analysis approaches such as the ability to narrow down monotherapy reports in FAERS and avoid confounding concurrent therapies and diverse indications. In contrast the who database provides a fixed interface without the option to consider monotherapy reports, or reports separated by indication.

## Discussion

Using the FAERS database, this study quantified the relationship between common osteoporosis drugs and the increased reporting of depression and anxiety. To further confirm our findings, we also performed an analysis with the WHO’s VigiAccess ADR data.

While two pharmacovigilance databases differed by the total number of reports and sources, the statistical calculations were in agreement and confirmed that alendronate had the strongest association with depression and anxiety among bisphosphonates (Figs. 1 and 3).

While risedronate the only other drug therapy to show a significant increase in depression reports both in FAERS and VigiAccess/WHO reports, the association was significant for anxiety in only WHO report analysis (Figs. 1 and 3).

Interestingly the association with depression and anxiety was significant for all the bisphosphonates in VigiAccess/WHO.

The data from the WHOs VigiAccess/WHO site appears to agree with the initial findings about alendronate, however it also points towards a possible class effect associated with the bisphosphonate class.

While the molecular mechanism of action which may explain the reported differences of depression and anxiety frequencies between alendronate and other bisphosphonates is not well understood, several unique pharmacological properties of alendronate have been described.

In a study by Wolffenbutell et al. a connection between calcium levels and psychiatric ADRs has been proposed^23^. In another study, alendronate was shown to inhibit Receptor type Tyrosine Protein Phosphatase S and E, PTPRS and PTPRE^24^. Coincidentally, a genome wide association study by Muglia et al. reported that the PTPRN, PTPRS and PTPRD receptors were significantly associated with major depression^25^. This poses a possible link between alendronate and its unique psychological adverse effects. The PTPRS receptor has also been shown to have function in synaptic organization, which, when impaired, could lead to a significant decline in neurotransmission as illustrated by studies of PTPRS knockout mice^26^. Bisphosphonates can cross the blood brain barrier^27–29^. Animal studies have shown alendronates' effect on inhibiting acetylcholinesterase enzymes in the frontal cortex, which could present another plausible mechanism for alendronates psychiatric effects^30^. Additionally, bisphosphonates as a class have long elimination half-lives, ranging from one to ten years, leading to compartmental accumulation and, therefore, potential long-term adverse reactions^31^. Bisphosphonates display multiphasic elimination due to their disposition into bone tissue which results in varying measurements of terminal half-lives depending on the type and sensitivity of assays^32,33^. The terminal half-life for alendronate was measured to be 10.5 years^33^, risedronate was 561 h^7^, zoledronate was 146 h^9^ and ibandronate was 37–157 h^8^. After bone disposition, the rate of elimination is reflective of the overall rate of bone turnover in the skeleton^33^ Based on this, it is estimated that after ten years of alendronate use, the amount of drug released from the skeleton daily approximately 25% of the amount absorbed in the gastrointestinal tract^6^. This resulting release of drug could cause potential for ADR even after treatment is terminated.

Psychiatric ADR frequency variability may also be attributed to dosing schedules. The recommended dosing schedule for alendronate is once daily to once weekly, for risedronate once weekly to once monthly, for ibandronate once every three months intravenously or once a month orally, and for zoledronate once a year intravenously (6–9). Interestingly, the frequency of dosing appears to align with the RORs reported for the VigiAccess data in order of least to most frequently taken leading to the highest magnitude for alendronate and lowest for zoledronate. More frequent dosing of drugs with longer half-lives may lead to larger cumulative side effects.

## Conclusion

In our study we calculated the reporting odds ratios of depression and anxiety in FAERS records of patients with osteoporosis on bisphosphonates and denosumab compared to teriparatide. We found that alendronate had the largest and statistically significant association with depression and anxiety out of all bisphosphonates. This association was supported by VigiAccess/WHO analysis. Controlled trials are necessary to adjudicate the clinical causality.

## Study limitations

The FAERS system is a collection of *voluntary* reports of adverse events and does not represent the true population of actual cases and adverse event frequencies due to underreporting and focus on more severe adverse events. While our study only looked at cases which used single drug therapy for a single indication, there could possibly be missing information regarding comorbidities and concurrent over the counter treatments. These could lead to inaccuracies in the calculated frequencies and odds ratios. The analysis was limited by the MedDRA terminology used in AE reporting which may not fully reflect specialized diagnostic scores. The detailed case narratives where not available due to privacy concerns.

The reported odds ratio calculation and 95% confidence intervals help to account for some of the uncertainties related to underreported confounding factors and small numbers of reports. While an association study cannot establish causation, the analysis of thousands of reports in the FAERS system acts as a tool to provide long term evidence for emerging adverse reactions long after a drug has passed clinical trials and entered the market.

## Data Availability

Data were obtained from the FDA Adverse Event Reporting System and the WHOs global database for ADRs and can be accessed at: https://www.fda.gov/Drugs/GuidanceComplianceRegulatoryInformation/Surveillance/AdverseDrugEffects/ucm082193.htm and http://www.vigiaccess.org/. The authors confirm that they did not have any special access privileges to these data.
